# Microbiome, Parkinson’s Disease and Molecular Mimicry

**DOI:** 10.3390/cells8030222

**Published:** 2019-03-07

**Authors:** Fabiana Miraglia, Emanuela Colla

**Affiliations:** 1 Department of Pharmacy, University of Pisa, via Bonanno 6, 56126 Pisa, Italy; miragliafabiana@gmail.com; 2 Bio@SNS Laboratory, Scuola Normale Superiore, Piazza dei Cavalieri 7, 56126 Pisa, Italy

**Keywords:** Parkinson’s disease, microbiota, molecular mimicry, microbiome, alpha-synuclein, curli, gut-brain axis, neurodegeneration

## Abstract

Parkinson’s Disease (PD) is typically classified as a neurodegenerative disease affecting the motor system. Recent evidence, however, has uncovered the presence of Lewy bodies in locations outside the CNS, in direct contact with the external environment, including the olfactory bulbs and the enteric nervous system. This, combined with the ability of alpha-synuclein (αS) to propagate in a prion-like manner, has supported the hypothesis that the resident microbial community, commonly referred to as microbiota, might play a causative role in the development of PD. In this article, we will be reviewing current knowledge on the importance of the microbiota in PD pathology, concentrating our investigation on mechanisms of microbiota-host interactions that might become harmful and favor the onset of PD. Such processes, which include the secretion of bacterial amyloid proteins or other metabolites, may influence the aggregation propensity of *αS* directly or indirectly, for example by favoring a pro-inflammatory environment in the gut. Thus, while the development of PD has not yet being associated with a unique microbial species, more data will be necessary to examine potential harmful interactions between the microbiota and the host, and to understand their relevance in PD pathogenesis.

## 1. Introduction

The term ‘microbiome’ refers to all the genomes of the microbial commensal community, hosted by our body. This ecosystem, which includes bacteria, archaeabacteria, fungi, protozoa and viruses, is defined as the microbiota [1]. In recent years, the importance of maintaining a healthy microbiota and its influence on human physiology has received much attention, since variations from this equilibrium have been linked to numerous human illnesses including neurodegenerative diseases. Because of this relevance, in 2007, the National Institutes of Health (NIH) launched the Human Microbiome Project with the purpose of identifying and elucidating the role of commensal microbial species in human health and diseases [1]. Surprisingly, this analysis showed that the entire microbiome includes more than 100 times the number of genes compared to the human genome [2], with a cell ratio of approximately 1:1 microbiota-host, and up to 1000 different species per individual [3]. 

In this review, we will focus our attention on the link between microbiota and Parkinson’s disease (PD). We will summarize recent findings connecting altered microbial composition (a phenomenon called dysbiosis) to PD and how molecular mechanisms of physiological interaction microbiota-host can become detrimental and may initiate or contribute to PD onset and pathogenesis. 

## 2. The Human Microbiome and Its Function

The abundance and diversity of the human microbiota are remarkable. Bacteria are the most prevalent members of this community, that reaches the highest density in the gut, particularly in the colon. Here, the most represented bacterial phyla are the Bacteroidetes and Firmicutes, while Actinobacteria, Proteobacteria, Verrucomicrobia and Fusobacteria are present at much lower levels [2,4,5,6,7]. Comparative computational studies have identified a “core” human microbiome, which is characterized by a set of common genes found in a specific habitat (e.g., gut, skin, oral mucosa, and vagina) in the majority of human subjects analyzed [5,7,8,9]. Thus, the *Streptococcus* genus, or more in general the phylum Firmicutes dominates the oral cavity and nares, whereas *Staphylococcus* (phylum Firmicutes), and less abundantly *Propionibacterium* and *Corynebacterium* (both phylum Actinobacteria), dominate the skin population. More interpersonal variation was found in other body habitats such as the vaginal mucosa and the gut [4,10,11]. 

Although the most diverse microbial community is present in the human GI tract, the microbiome regulates human physiology well beyond intestinal function. This regulation is executed through a myriad of different homeostatic, immunologic and metabolic activities, but because of the high redundancy in microbial species, it is still difficult to determine exactly which strain is responsible for each function. The importance of microbiota-host interactions is highlighted by the observation that germ-free (GF) animals that are born and housed without exposure to any microbe show multiple systemic deficiencies including an immature immune system and a not fully developed and functional gastrointestinal (GI) tract [12]. Interestingly, some of these impairments, such as a more functional intestinal immunity, can be recovered in GF animals after recolonization [12,13].

In the gut, the microbiota contributes to the growth, regulation and differentiation of intestinal epithelium of the small and large bowels [14], modulates GI motility and promotes normal development of the enteric nervous system (ENS) [15,16,17], regulates the integrity and fortifies the mucosal barrier, stimulates angiogenesis and mediates postnatal intestinal maturation [14,18]. The microbial community reinforces the intestinal barrier creating an adverse environment for potential pathogenic bacteria by subtracting nutrients and producing antimicrobial peptides which are able to counteract them [10,19]. 

As for the immune system, close microbiota-host interconnection has resulted in the development of several molecular mechanisms that allow the host defense to learn to tolerate the commensal community, while at the same time to function properly. In this way, both the innate and adaptive responses appear to be influenced and programmed by the presence of the microbiota. For instance, the innate immune response has developed a system of protein receptors to recognize general microbe-associated molecular patterns (MAMPS) that are similar across bacteria species, such as components of the bacterial cell wall [lipopolysaccharide (LPS) and peptidoglycan] and flagellin [20]. This family of receptors, that includes transmembrane Toll-like receptors (TLRs) and intracellular Nod-like receptors (NLRs), is the first line of defense against invading microbes. TRLs activation leads to the initiation of the innate immune response through the induction of a series of transcription factors such as NF-κB, AP-1, Elk-1, CREB, STAT involved in the regulation of pro- or anti-inflammatory cytokines and chemokines production [21], while NLRs can oligomerize forming the inflammasome and activating, in turn, caspase-1 and the pro-inflammatory cytokines cascade [22,23]. TLRs are expressed on immune cells as well as neurons. In the gut, where the intestinal mucosa is intimately associated with the ENS, TLRs are not only sensors of microbial invasions, but are also important in mainting gut homeostasis and neurochemical communication with the ENS. For instance TLR2 or TLR4 ablation in mice impairs the structural and functional integritiy of intestinal mucosa, alters gut motility, and reduces the number of myenteric neurons and the production of neurotrophic factors [15,16]. 

In the acquired response, the gut microbiome is known to shape the differentiation and function of anti-inflammatory regulatory T cells [24,25] and to facilitate the switching of B cells to produce and secrete IgA [26]. 

Microbial species can provide nutrients from substrates that are otherwise indigestible by the host, such as dietary fibers, from which the host obtains an important energy source like small chain fatty acids (SCFAs). SCFAs are important in maintaining a regular colon homeostasis, bowel motility and normal, intestinal cellular growth, lowering in this way the risk for cancer [27]. The microbiota is also responsible for secreting vitamins such as vitamin B12 synthetized by *Lactobacillus reuteri* or vitamin B2 produced by *Bacillus subtilis* and *Escherichia coli* [28] and to modulate lipid and bile acids metabolism [29,30]. Additionnally, the microbiome is known to participate in the metabolism of environmental chemicals such as heavy metals and arsenic, pollutants such as polycyclic aromatic hydrocarbons, and drugs and medications including the activation of prodrugs such as in the case of azo drugs, resulting in the release of sulphanilamide (for a review see [31]).

Thus, while what we have illustrated are only few examples of how the resident microbiota can influence host homeostasis, it is important to mention that this is a bidirectional dialogue where the host has evolved cellular mechanisms which are able to regulate microbiota composition by favoring the growth of beneficial species while fighting pathogens. For example, a specific type of host innate lymphoid cells, ILC3 which are located in the lamina propria of the intestinal mucosa, can release cytokines, which are able to activate, in turn, the epithelial cells to produce and secrete antimicrobial peptides such as defensis and mucins that can regulate and influence microbiota composition [32]. In addition, the shedding of fucosylated surface proteins of intestinal epithelial cells, which is induced by ILC3 signals, can be used as an energy supplement for sustaining resident commensal bacteria [33].

Multiple elements, such as environmental and host factors like age, human health and diet, are crucial in determining an intra-individual and an inter-individual variability of the microbiota. In particular, the prolonged use of antibiotics has been shown to promote the development of multi-drug resistance bacterial strains, the loss of colonization resistance and the domination by pathogenic bacteria and long-term changes in the composition of microbial residents [34]. For instance, a longitudinal study in a small cohort of human subjects demonstrated how the use of ciprofloxacin had a long-term reduction in bacterial diversity, even after 6 months from the completion of the treatment [35]. 

Alterations in the maturation or in the composition of the microbiota, particularly in childhood, can increase the risk of an over-reactive immune system, and have been connected to the onset of several pathologies such as asthma, allergies, autoimmune diseases, inflammatory bowel disease (IBD), Crohn’s Disease, diabetes, atherosclerosis, cardiovascular disease, multiple sclerosis and rheumatoid arthritis [36]. At the same time, modifications in the microbiome have been associated with metabolic syndrome, such as obesity and obesity-related disorder [5,37], or increased risk of colorectal carcinoma [38]. Thus, while we are still learning the profound interconnections of human physiology with the resident microbial community, strategies aimed at restoring a healthy and balanced microbial population have been shown to successfully treat some human pathologies. One example is intestinal colitis induced by recurrent *Clostridium Difficile* infection (RCDI). Fecal microbial transplantation, a procedure involving the oral or rectal transfer of fecal samples obtained from healthy donors, was able to cure more than 90% of patients with RCDI after simultaneous suspension of antibiotic therapy [39]. Because of its high success rate, fecal microbial transplantation has been applied to other types of GI inflammatory illnesses such ulcerative colitis and IBD [40]. Although concerns over the long-term consequences in meddling with the resident microbial species are still present, therapies based on microbial transfer or on treatment with bacterial metabolites seem to be a promising tool in fighting human diseases and antibiotic resistance. 

### Microbiome and the Gut-Brain Axis

Alteration of the intestinal integrity and activity of the microbiota can influence brain function. The gut–brain axis is a bidirectional communication system that uses neural, endocrine and immunological signals. Neural information is thought to travel along vagal or spinal innervation between the CNS and the gut. The microbiota can send several signals directly to the CNS or indirectly, via the enteric nervous system (ENS) through the synthesis of neurotransmitters or neurochemical-like precursors. Multiple examples have been found of such activity like GABA synthesis by *Lactobacillus* and *Bifidobacterium* species; noradrenaline by *Escherichia*, *Bacillus* and *Saccharomyces* species; serotonin by *Candida*, *Streptococcus*, *Escherichia* and *Enterococcus* species; dopamine by *Bacillus* species and, ultimately, acetylcholine by *Lactobacillus* [41]. In addition *Bifidobacterium infantis* has been shown to increase the plasma level of tryptophan, a key precursor of serotonin [42]. At the same time, it has been found that SCFAs can also, among other functions, stimulate the production of serotonin by intestinal enterochormaffin cells [43], control maturation of microglia in the CNS [44], modulate the ENS activity by interacting with G-protein coupled receptors such as GPR41 and GPR43 [29,45] and by mediating epigenetic modulation through histone deacetylation [46]. 

Immune signaling as part of the gut and brain communication system is mediated by the release of cytokines. It is thought that such a mechanism, through the induction of IL-1 and IL-6, can stimulate the hypothalamic-pituitary-adrenal (HPA) axis to produce cortisol, a key hormone of the stress response. Activation of the HPA axis has been shown to influence gut resident microbial population. For instance, mice subjected to water-avoidance stress or neonatal stress produced by maternal separation showed alterations in the microbiota composition [47,48]. GF mice subjected to prolonged restraint stress showed an over-reactive HPA response, with increased release of corticosterone and ACTH in plasma, that could be restored to a normal level by recolonization with *Bifidobacterium infantis* [49]. Although with a genetic basis, autism spectrum disorders have also been associated with a disruption of the gut-brain axis. Altered composition of the gut microbiota [50,51], severe GI dysfuction [52] and increased intestinal permeability [53] have been shown in autistic children, while dietary restriction, such as a gluten-free diet, was found to ameliorate GI symptoms as well as their social behavior [54]. In agreement with this observation, the microbiota has been found to modulate social or stress-correlated human behavior. Gut microbial reconstitution improved social and behavioral deficits induced in mice by maternal obesity [55], whereas recolonization with *Lactobacillus reuteri* was sufficient to recover metabolic function and behavioral impairment in chronically depressed mice [56].

Additionally, gut microbial richness and diversity, as well as the integrity of the intestinal and blood brain barrier have been associated with healthy aging while any perturbation of this balance could lead to a systemic pro-inflammatory condition and influence, in turn, the development of neurological disorders including neurodegenerative diseases [57].

## 3. Origin of PD and Alpha-Synuclein Transmission

PD is a neurodegenerative disorder that has long been thought to typically affect the motor system, in particular the nigra-striatal pathway, characterized by dopaminergic neuron loss in nigra-striatal area and accumulation of intraneuronal proteinacious inclusions named Lewy Bodies (LBs) and Lewy neurites made of alpha-synuclein (αS) [58]. This dopamine-centric view has been challenged in the last decade by Braak and colleagues that described finding in PD subjects as well as healthy controls, LBs in remote locations, nonclassically linked to PD, such as the ENS, the olfactory bulbs and dorsal motor nuclei of the vagus nerve [59]. According to these observations, Braak and colleagues theorized a staging of PD pathology, whereby initial accumulation of LBs would take place in neuronal cells of the olfactory bulbs and/or in the ENS and then spread in a retrograde or ascending manner following anatomical connections to the brainstem and the forebrain [60]. This staging theory correlated well with the fact that up to 70 % of PD patients had a recurrent history of GI dysfunctions and/or complained of anosmia years before being diagnosed [61]. After Braak’s first observation in 2003, several groups showed the presence of LBs outside the CNS, with a higher frequency in the vagus nerve, sympathetic ganglia, ENS, submandibular glands and in the endocrine system [62,63,64]. Further, in support of the Braak’s staging thesis, αS ‘s ability to act as prion protein and to transfer its pathogenic template has been shown in cellular and animal models of PD [65,66,67,68]. In this context, it was evident that fibrils or oligomers of αS were more efficient than the native monomer in seeding endogenous aggregation. Moreover, intracerebral injections in wild-type mice of human αS fibrils or brain extracts from PD patients and diseased transgenic mice showed that toxic conformations of αS can propagate within the CNS following anatomical connections by transferring their pathogenic template to endogenous mouse αS [69,70,71,72]. More recently, multiple conformations of αS (oligomers, ribbons, fibrils) were all shown to be pathogenic, giving rise to a different kind of PD-like pathology and confirming αS as a true infectious agent [73]. Propagation of toxic αS aggregates from the peripheral nervous system (PNS) to the CNS was confirmed after injection of αS synthetic fibrils into hind limb muscle or in the intestinal wall [74,75]). In the latter, active axonal transport of αS aggregates from the ENS to the midbrain was seen following the vagus nerve connection, in open support to Braak’s staging. Similar findings were shown in a different PD animal model, where the accumulation of αS inclusions in the ENS, in the dorsal motor nucleus of the vagus nerve, in intermediolateral nucleus in the spinal cord and in the brainstem, was induced by rotenone treatment [76]. Again, partial vagotomy was able to delay αS spreading, indicating vagal innervation as a probable route of dissemination of αS toxic forms [77]. Notably, in the same study, partial resection of the mesenteric nerve that innervates, among others, the distal colon, also delayed αS dissemination. This suggests that in addition to vagal innervation, other routes may contribute to αS propagation (for a review [78]). Finally, in humans, truncal vagotomy has been shown to exert a protective role in developing PD [79]. 

In support of a role for the ENS in the development of PD, the presence of gut inflammation and increased intestinal permeability has been demonstrated in PD patients [80,81]. This inflammatory condition was associated with increased LPS-binding protein in plasma, upregulation of pro-inflammatory cytokines production in the colon and glial cells, and with activation and structural reorganization of the epithelial barrier with downregulation of specific tight junction proteins such as occludin [82,83,84]. Interestingly, intraperitonal administration of a low dosage LPS induces αS deposition in the colon of treated rats concomitant to increased intestinal permeability [85]. In addition, both monomer and aggregated forms of αS were shown to have chemotactic abilities toward isolated human neutrophils and monocytes cultures, and to be able to promote dendritic cells maturation in vitro [86].

Thus, the accumulation of αS inclusions in the gut may contribute directly to local inflammation, exacerbating gut dysfunction in clinical and preclinical PD. What is causing initial αS conversion to its toxic conformation is still unknown, although cellular stress such as oxidative insults, inhibition of the protein degradation mechanism, endoplasmic reticulum stress or specific cellular environment have been assumed to contribute to stabilizing toxic conformations of αS [87,88,89,90]. However, in the gut, because of the augmented intestinal permeability and the increased translocation of bacterial products across the intestinal wall, a harmful interaction with pathogens or their metabolites may provide new clues on the αS path to toxicity. 

## 4. Microbiome and PD

Changes in the microbial resident population with alteration in the production of microbial metabolites have been observed in human PD subjects. When comparing the human microbiome samples from healthy controls versus PD patients, significant dysbiosis was observed in PD that correlated to a change in the relative abundance of certain bacterial genus or species, rather than the appearance or disappearance of a particular microbial population. In particular, several studies reported an increase in the relative abundance of genus *Lactobacillus* [91,92], *Bifidobacterium* [90,91], the family of *Verrucomicrobiaceae* [91,92,93], including the genus *Akkermansia* in PD [93,94,95,96], while others showed decrease in the level of genus *Faecalibacterium* [93,94,96,97], *Coprococcus* and *Blautia* [91,93,98], *Prevotella* [94] and other genus within the Prevotellaceae family [91,97]. Other studies showed how microbiota alteration such as increases in abundance in *Christensenellaceae* or *Oscillospira* families correlated to an augmented susceptibility to develop PD or to a specific disease stage [92,95], suggesting that such modifications in the microbiome population could be used for early diagnosis. The obstacles in finding such a biomarker, however, lie in the extreme variability and poor reproducibility in data analyses which were undertaken during microbiome assessment studies. DNA isolation methods, the recruitment of cohorts from non-homogenous PD populations that include differences in diet, age, severity of the pathology and PD stage and treatment with PD medications, are all factors that can affect the microbiome [91,94,96], and are probably responsible for early published data that seemed to contradict itself [92,93,95,97]. Better, standardized methods in sampling collection and in DNA extraction are necessary in order for these studies to be more conclusive and informative. 

In agreement with this observation, the role of unclassified bacteria, estimated to represent about 40% of the gut microbiome but usually present in low abundance (below 1%), is still unclear. Interestingly, it has been shown how one of these species, the abundance of which was found to be increased in PD patients, operational taxonomic unit 469, expresses an endonuclease with a αS-like domain [98]. Thus, although present in extremely low abundance, unclassified bacteria species might exhibit specific types of interactions with the host that can significantly modify human pathophysiology.

A link between microbial dysbiosis and neurodegeneration has also been shown in genetic and pharmacological animal models of PD. In a transgenic mouse line for human αS, GI and motor deficit, αS deposition and neuroinflammation were ameliorated by GF housing conditions or antibiotics treatment [96]. More remarkably, however, such improvements were negatively counteracted by recolonization of the same line, housed in GF cages, with fecal, human microbiota from PD patients. At the same time, treatment with human microbiota from healthy controls had no effect, suggesting that a specific interaction with the host may influence the onset of a genetic-based trait or dysfunction. Because a healthy intestinal development requires microbiota colonization and robust antibiotic treatments can also influence the host physiology [99], data on GF animals should be taken cautiously, and such massive effects should be first investigated for their relevance to their host’s physiology. 

Changes in microbiome composition were also observed in pharmacologically-induced PD models, such as after exposure to rotenone [12,100] or MPTP [101,102]. Some of the variations in abundance reported such as increases in *Bifidobacterium* and *Lactobacillus* genus and a decrease in *Prevotellaceae* after rotenone treatment or the increase in *Enterobacteriaceae* in MPTP-treated mice were similar to what has been observed in human PD cases [103,104]. Nevertheless, apart from dysbiosis, both genetic [100,105] and toxin-induced PD models [99,106,107] showed a complex intestinal phenotype, including constipation, gut inflammation and increased intestinal permeability, suggesting how changes in the microbial resident populations are directly or indirectly related to a more complex scenario, involving a strict interaction with the host. 

Thus, as mounting evidence supports a role for the microbiota in the regulation of human behavior and neuronal functions, concerns arise about possible dentrimental interactions with the commensals and its consequences in terms of the development of neurological disorders. But how? Is dysbiosis the result of a dysfunctional GI tract or is it the causative agent? And if it is the causative agent, what are the implications in terms of PD pathogenesis? 

## 5. Mechanisms of Molecular Mimicry 

The concept of molecular mimicry was originally described to explain structural similarities in biology that developed in response to evolutionary pressure. Microorganisms mimicking protein structures in their host may have an advantage in escaping immune detection. Such similarities may be due to aminoacid sequence homology, but also to shared nucleotide sequences or protein structures. For instance, autoimmunity is thought to be the result of the development of antibodies targeting proteins of pathogens whose protein sequence is similar to that of the host. Together with fighting pathogen infection, those antibodies will also attack the host, causing disease. This seems to be the case of rheumatic fever, where streptococcal M protein and streptococcal group A carbohydrate epitope, N-acetyl glucosamine, bear strong sequence resemblance to cardiac myosin. An immune response against those bacterial epitopes can go awry and attack the cardiac valve, causing chronic rheumatic heart disease [108]. More recently, the discovery of structural similarities between certain plant viral RNAs with human microRNAs has suggested that homology in nucleotide sequences may represent a survival advantage for certain viruses that can, in this way more easily override and take over the host cellular metabolism. For instance, certain viral RNAs can mimick microRNA functions in the host, and thus, influence host gene expression. An example of such a mechanism is provided by the infection of *Herpes virus saimiri*. This virus, that infects primates, expresses two non-coding RNAs that are able to bind and promote the degradation of the host miR-27, modifying the expression in this way of miR-27 target genes [109].

Besides homology in aminoacid or nucleotide sequences, molecular mimicry can also be dictated by the existence of tertiary protein structure similarities. One example of molecular mimicry is the amyloid conformation in protein folding, a structure that is highly conserved through evolution and widely distributed among living organisms, including bacteria. The human microbiota can produce and secrete extracellular amyloid proteins that are responsible for enhancing bacterial adhesion, colonization and biofilm formation, but also tissue invasion and infectivity [110]. Bacterial amyloids secreted by Gram-positive and Gram-negative bacteria are long fibers, extending from the cell surface, with a specific and highly controlled pathway of assembly [111,112]. In humans, despite the pathogenic accumulation of amyloid proteins in neurological diseases, the formation of functional amyloids has been implicated in synaptic plasticity and memory storage [113], melanin biosynthesis [114] and innate immune response [115]. 

Thus, the production of amyloid proteins has a clear functional role in physiology, evolution and adaptation to the environment. Nevertheless, the ability of certain amyloid-forming proteins, such as PrP^Sc^, Tau and αS to act as a prion and transfer their pathogenic template [69,70,71,72,116,117], amylogenic cross-seeding demonstrated for aggregates-prone proteins such as PrP^Sc^, αS, Aβ and Tau [118,119,120,121], dysbiosis and GI dysfunction found in PD patients [92,95] and formation of LBs in the olfactory bulbs and the ENS in humans [59,62,64], areas that are majorly exposed to the environment and consequently colonized by the microbiota, are all mounting evidence suggesting that bacterial amyloids might also be involved in detrimental interactions with their host. For instance, *E. coli* and *Salmonella typhimurium* secrete an extracellular amyloid protein called curli, involved in colonization and biofilm development [111,122]. Curli fibers can be stained by Congo red and Thioflavin T [123,124], dyes routinely used to visualize proteinacious insoluble plaques and protein aggregation in neurodegenerative diseases [125,126], and have been found in the GI tracts of humans [127]. 

## 6. Evidence of Molecular Mimicry in the Pathogenesis of PD

Can microbiota-derived amyloids induce misfolding of aggregation-prone proteins in the host? Very little evidence has been published supporting this hypothesis. Curli and two other protein amyloids, silk and Sup35, produced respectively from *Bombyx mori* and *Saccharomyces cerevisiae*, were found to accelerate inclusions formation of amyloid protein A (AA) in a murine experimental amyloidosis model after systemic injection [128]. Mass-spectrometry analysis of in vivo-isolated AA aggregates revealed no trace of exogenous fibrils, suggesting that pathogenic nucleation was, in this case, an initial event. In contrast, in vitro experiments have shown how curli fibers can affect both nucleation as well as elongation steps in the cross-seeding process of other amyloid-prone proteins such as fragments of prostatic acid phosphatase (PAP_248–286_), islet amyloid polypeptide and *A*β_1−40_ [129]. Moreover repeated oral administration of curli-producing bacteria, but not its corresponding curli-deficient mutant line, to aged Fischer 344 rats, a wild-type line known to accumulate intestinal αS inclusions with aging [130], or to a *C*. *elegans* line overexpressing *α*S, induced increased neuronal αS deposition in both the gut and the brain tissues [131]. In addition, aged Fischer 344 rats developed microgliosis and astrogliosis associated with elevated levels of TLR2, IL-6 and TNF in the brain [130]. The TLR2/1/CD14 heterocomplex recognizes the β-sheet secondary structure of curli and activates NF-kB, eliciting the production of pro-inflammatory chemokines and cytokines including IL-8, IL-6, IL-17A and IL-22 [132,133,134,135]. In addition to the TLR2/1/CD14 heterocomplex, curli is also recognized by the NLRP3 inflammasome, which leads to the activation of caspase-1/11 and the maturation of pro-IL-1β to IL-1β [136]. 

Thus, besides a direct pathogenic cross-seeding between amyloids of different organisms, bacterial amyloids are recognized as MAMPS, and can directly stimulate and prime the host’s immune response, contributing to pro-inflammatory conditions in the gut and, in turn, favoring an environment that may facilitate protein aggregation, cellular dysfunction and death. In addition, because inflammatory signals may be sent to the brain, directly through cell infiltration or indirectly, it is possible that human *α*S might be recognized as a MAMP mimicking bacterial amyloids. For instance, in the brain, neuronal-secreted oligomeric *α*S can bind to TRL2 on microglia and downstream activate production of TNF and IL-1β [137,138]. Furthermore, TRL4 expression on microglia seems to be required for soluble and aggregated *α*S phagocytosis [139], and TRL-4 KO mice are more resistant to rotenone-induced neuroinflammation and neurodegeneration [81]. Although it is not clear if *α*S antigenic potential needs a primed immune system in the gut, because of the importance of neuroinflammation and TRLs in PD, therapeutic intervention targeting downregulation of TRLs signaling is under review [140], and could represent a unique strategy to counteract both gut and brain inflammation. 

Apart from exogenous amyloids, other bacterial metabolites such as LPS, a TLR4 ligand, or SCFAs like butyrate may be responsible for toxic microbiota-host interactions. For instance, systemic injections of LPS in wild-type mice is commonly used as a pharmacological paradigm of PD. Through TNFα activation, the administration of LPS causes chronic peripheral and central neuroinflammation with microglia activation and production of pro-inflammatory cytokines, resulting ultimately in damaging dopaminergic neurons in the substantia nigra [85,141,142]. In addition, the repeated oral administration of *Proteus Mirabilis* or its derived LPS to MPTP-treated or young wild-type mice was sufficient to induce a PD-like phenotype including motor deficit, dopaminergic neuronal loss in the nigra, brain and gut inflammation with disruption of the intestinal epithelial barrier and formation of αS inclusions in the brain and in the colon [105]. In a similar way, treatment with a mixture of the SCFAs (acetate, propionate, and butyrate) of αS transgenic mice housed in sterile conditions exacerbated neuroinflammation, onset of motor dysfunction and αS aggregation in PD-affected brain regions [100]. Interestingly, SCFAs did not favor αS amyloid formation in vitro, indicating that inclusions formation in vivo was not the result of a direct molecular interaction with such bacterial lipids. At the same time, the administration to SCFAs-treated αS mice of minocycline, a drug that targets TNFα activation, reduced αS aggregation and improved motor function, suggesting the importance of systemic inflammation in mediating toxicity in the interaction host-microbiota.

Besides molecular mimicry mechanisms involving bacteria, the increased accumulation of antibodies against specific viral infections such as *herpes simple-1* and *Epstain Barr virus* (EBV) has been detected in blood samples of PD patients [143,144]. Interstingly, in both cases, antibodies targeting viral proteins showed cross-reactivity to human αS, and it was shown how the C-terminal of LMP1, a late membrane protein of EBV, bears a strong aminoacid sequence homology to αS C-terminal. 

Thus, a direct and/or indirect interaction with a specific virus or bacterial metabolites that goes awry, possibly mediated by a systemic pro-inflammatory condition, could ultimately contribute to the initiation or acceleration of the onset of PD.

## 7. Conclusions

In the last decade, research on commensal resident microbial species has elucidated the importance of the microbiota on human physiology and disease. While some human illnesses may be more directly caused by dysbiosis and colonization of pathogenic organisms, for example, enteric infections and IBD, the connection with neurological disorders, including neurodegenerative diseases, is less clear. Dysbiosis has been shown in PD patients, but there is no evidence thus far that links PD pathology to the presence of a specific microbial species. What makes αS initially transit to a toxic conformation is still largely unknown. Nevertheless, multiple evidence, including the fact that LB deposition may be initiated in locations more which are directly in contact with the external environment and that certain microbial species can secrete amyloid proteins, supports the hypothesis that a harmful influence of the microbial community might be implicated in αS toxic conversion and spreading. Future research should more directly address questions about the bacterial transmission of pathogenic amyloid conformations to the host and, at the same time, take into consideration individual susceptibility, as dictated by the environment and by the subject’s genotype, in facilitating αS spreading and the onset of neurodegeneration. 
